# Combined targeting of AKT and mTOR synergistically inhibits proliferation of hepatocellular carcinoma cells

**DOI:** 10.1186/1476-4598-11-85

**Published:** 2012-11-20

**Authors:** Nicole Grabinski, Florian Ewald, Bianca T Hofmann, Katharina Staufer, Udo Schumacher, Björn Nashan, Manfred Jücker

**Affiliations:** 1 Center for Experimental Medicine, Institute of Biochemistry and Signal Transduction, University Medical Center Hamburg-Eppendorf, Martinistr. 52, Hamburg 20246, Germany; 2 Department of Hepatobiliary and Transplant Surgery, University Medical Center Hamburg-Eppendorf, Martinistr. 52, Hamburg, 20246, Germany; 3 Center for Experimental Medicine, Department of Anatomy and Experimental Morphology, University Cancer Center, University Medical Center Hamburg-Eppendorf, Martinistr. 52, Hamburg, 20246, Germany; 4 Present address: Department of Internal Medicine III, Division of Gastroenterology and Hepatology, Medical University of Vienna, Vienna, Austria; 5 Present address: Department of General, Visceral and Thoracic Surgery, University Medical Center Hamburg-Eppendorf, Hamburg, Germany

**Keywords:** Hepatocellular carcinoma, RAD001, MK-2206, Proliferation, AKT, AKT isoform kinase assay

## Abstract

**Background:**

Due to the frequent dysregulation of the PI3K/AKT/mTOR signaling pathway, mTOR represents a suitable therapeutic target in hepatocellular carcinoma (HCC). However, emerging data from clinical trials of HCC patients indicate that mTOR inhibition by RAD001 (Everolimus) alone has only moderate antitumor efficacy which may be due to the feedback activation of AKT after mTOR inhibition. In this study, we analyzed the effects of dual inhibition of mTOR and AKT on the proliferation of HCC cell lines. In addition, we measured the feedback activation of each of the AKT isoforms after mTOR inhibition in HCC cell lines and their enzymatic activity in primary samples from HCC patients.

**Methods:**

The activation status of specific AKT isoforms in human HCC samples and corresponding healthy liver tissue was analyzed using an AKT isoform specific *in vitro* kinase assay. AKT isoform activation after mTOR inhibition was analyzed in three HCC cell lines (Hep3B, HepG2 and Huh7), and the impact of AKT signaling on proliferation after mTOR inhibition was investigated using the novel AKT inhibitor MK-2206 and AKT isoform specific knockdown cells.

**Results:**

AKT isoforms become differentially activated during feedback activation following RAD001 treatment. The combination of mTOR inhibition and AKT isoform knockdown showed only a weak synergistic effect on proliferation of HCC cell lines. However, the combinatorial treatment with RAD001 and the pan AKT inhibitor MK-2206 resulted in a strong synergism, both *in vitro* and *in vivo*. Moreover, by analyzing primary HCC tissue samples we were able to demonstrate that a hotspot mutation (H1047R) of PI3KCA, the gene encoding the catalytic subunit of PI3K, was associated with increased *in vitro* kinase activity of all AKT isoforms in comparison to healthy liver tissue of the patient.

**Conclusion:**

Our results demonstrate that dual targeting of mTOR and AKT by use of RAD001 and the pan AKT inhibitor MK-2206 does effectively inhibit proliferation of HCC cell lines. These data suggest that combined treatment with RAD001 and MK-2206 may be a promising therapy approach in the treatment of hepatocellular carcinoma.

## Background

Hepatocellular carcinoma (HCC) is the third leading cause of cancer mortality worldwide, with an increasing incidence in the United States and Europe [1,2]. Only 30-40% of patients are amenable to potentially curative therapies, such as surgical resection, because of the often advanced stage of disease at the time of diagnosis. Today, the multi-kinase inhibitor sorafenib is the only systemic therapy to improve survival in these patients [3]. However, prognosis of advanced HCC remains poor, and new effective therapeutic strategies are urgently needed.

The PI3K/AKT/mTOR signaling pathway is a promising target with respect to its frequent dysregulation in hepatocellular carcinoma and its central role in regulating cell proliferation, migration, survival and angiogenesis [4,5]. Aberrant mTOR signaling has been detected in up to 48% of hepatocellular carcinoma, and a correlation between poor outcome and mTOR signaling activation has been shown [6]. Phosphorylation of AKT at S473 was detected in up to 71% of HCC samples, and associated with invasion, metastasis, and vascularization of HCC [7].

Several inhibitors targeting mTOR are tested in clinical trials at present [8]. Clinical trials revealed that the rapamycin derivative RAD001 (Everolimus) is sufficient to improve the overall survival of patients with metastatic renal carcinoma and subependymal giant cell astrocytoma [9,10]. Emerging data from clinical trials of HCC patients indicate that RAD001, even well tolerated, has only moderate antitumor efficacy in HCC patients [11,12]. A negative feedback loop resulting in the activation of AKT following mTOR inhibition has been observed in a variety of cancer cell lines and human tumor samples of colon and breast cancer [13]. Since the antitumor efficacy of rapalogues in patients is modest, activation of AKT as a central regulator of cell growth is a potentially unfavorable event resulting from cancer treatment with mTOR inhibitors [14]. AKT feedback is thought to be mediated by inhibition of p70S6 kinase activity, resulting in an increase in IRS-1 (insulin receptor substrate 1) expression [15]. Since it has been demonstrated that cells lacking IRS-1 also show increased AKT phosphorylation following mTOR inhibition, other mechanisms of feedback activation have been proposed [16]. An increase in the phosphorylation of receptor tyrosine kinases, i.e. EGFR, HER2, HER3 among others, following treatment with rapamycin was demonstrated [17] and may represent an IRS-1-independent way of increased AKT signaling.

The serine/threonine kinase AKT is a key player of the regulatory network of the cell and affects virtually all cellular activities, including growth, survival, movement, differentiation and metabolism [18,19]. Currently, three mammalian isoforms of AKT (AKT1/PKBα, AKT2/PKBβ, and AKT3/PKBγ) have been identified [20,21]. The isoforms share a high degree of structural homology with human AKT1 having 81 and 83% amino acid identity with AKT2 and AKT3, respectively. Although there is evidence for partial functional overlap of the AKT isoforms, studies on isoform specific knockout mice revealed their distinct functional roles underlined by diverse signaling cascades, thereby controlling cell growth, metabolism, cell proliferation and survival [22,23]. Specific functions of each isoform have also been proposed in human carcinoma cell lines and mouse models [24-26].

In this study, we analyzed the AKT feedback activation following RAD001 treatment in three HCC cell lines with different AKT isoform expression levels. For the first time differential changes in AKT isoform activity following RAD001 treatment were documented. Furthermore, the importance of AKT isoform specific signaling following mTOR inhibition has been investigated by combination of the mTOR inhibitor RAD001 either with the new orally active allosteric pan AKT inhibitor MK-2206 [27], or AKT isoform specific knockdown cells. Here, we demonstrate that inhibition of mTOR and AKT in combination acts synergistically on cell proliferation of HCC cells. Our results suggest that dual targeting of mTOR and AKT might be a new promising therapeutic approach in the treatment of hepatocellular carcinoma.

## Results

### Feedback activation of AKT following mTOR inhibition with RAD001 is concentration- and time-dependent

**Figure 1:**
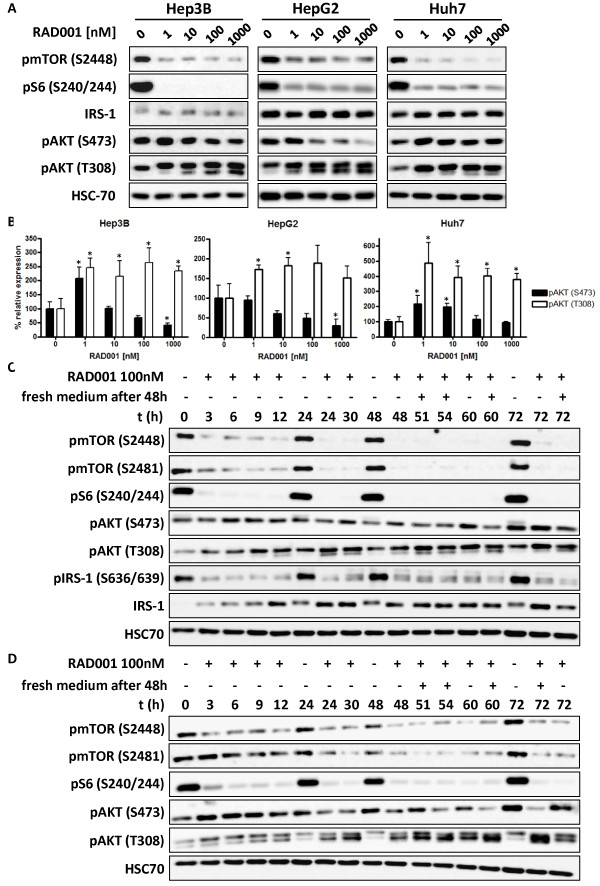
**Feedback activation of AKT after mTOR inhibition is time- and dose dependent.** (**A**) HCC cell lines Hep3B, HepG2 and Huh7 were treated for 24 h with increasing concentrations of RAD001 as indicated. Phosphorylation status of mTOR and AKT was analyzed by Western blot with phospho specific antibodies. (**B**) Dose dependent expression of pAKT (S473) and pAKT (T308) after 24 h treatment with RAD001 was quantified from three independent experiments as shown in A. Columns, mean percentage of three independent experiments; bars, SD, * p < 0.05. (**C**) Huh7 and (**D**) HepG2 cells were treated with 100 nM RAD001 up to 72 h, and cell lysates were prepared at the indicated time points. Where indicated, medium was removed after 48 h and replaced by fresh, 100 nM RAD001 containing medium. HSC70 served as loading control.

In order to investigate feedback activation of AKT following mTOR inhibition we analyzed the activation of the PI3K/AKT/mTOR pathway in the three HCC cell lines Hep3B, HepG2 and Huh7. All HCC cell lines showed a constitutive activation of the PI3K/AKT/mTOR pathway as demonstrated by phosphorylation of AKT (S473 and T308), mTOR (S2448), representing mTORC1 activity [28], and pS6 (S240/S244) which is a downstream substrate of mTORC1 and S6-kinase (Figure 1A). Treatment of the HCC cell lines with various concentrations of RAD001 resulted in a marked suppression of phosphorylation of mTOR (S2448) and the downstream S6 protein (S240/244) (Figure 1A). Interestingly, we observed a differential feedback activation of AKT in a concentration-dependent manner after treatment with RAD001 for 24 hours with respect to phosphorylation of AKT at residues S473 versus T308 (1B). The feedback phosphorylation at S473 showed a peak level at 1 nM (Hep3B and Huh7) and only a lower or even no increase at all at higher concentrations of RAD001. In contrast, feedback phosphorylation of T308 was observed at low and high concentrations of RAD001 (Figure 1A, B).

Because we did not observe an increase in the phosphorylation of AKT at S473 in HCC cells after treatment with a higher concentration of 100 nM RAD001 at 24 hours (Figure 1B), we performed a more detailed time kinetic from 3 up to 72 hour (Figure 1C and D, Additional file 1: Figure S1). No significant time dependent change in pAKT (S473) expression was observed in Hep3B cells at 100 nM RAD001 (Additional file 1: Figure S1). Surprisingly, a short term increase of pAKT (S473) expression was evident in both Huh7 (Figure 1C) and HepG2 (Figure 1D) cells with a peak between 3 to 12 h after treatment with RAD001. The basal pAKT (S473) expression level was reestablished after 24 h and no further increase was observed at 48 h post-treatment. The decrease of pAKT (S473) represents inhibition of mTORC2 activity [29]. This is in line with previous studies showing suppressed mTORC2 assembly after prolonged incubation of cells with allosteric mTOR inhibitors [30]. In the case that the culture medium was replaced after 48 h with medium containing fresh RAD001, subsequent AKT feedback phosphorylation on S473 was absent or only weakly detectable 3 to 6 h after the addition of new RAD001 (Figure 1C and D). Conversely, there was a clear time-dependent increase in pAKT (T308), reaching its maximum after >30 h of incubation. While cells treated one time only with RAD001 show elevated levels of pAKT (S473) after 60 and 72 h, no increase of pAKT (S473) expression was observed after 60 and 72 h in cells re-treated with fresh culture medium after 48 h (Figure 1C and1D). This late increase in pAKT (S473) might be due to degradation of RAD001 in culture medium, thereby possibly relieving mTORC2 complexes of RAD001-mediated inhibition which was described before [31]. All results were confirmed in at least two independent experiments.

### Differential and isoform specific activation of AKT1, AKT2 and AKT3 during feedback activation after mTOR inhibition

**Figure 2:**
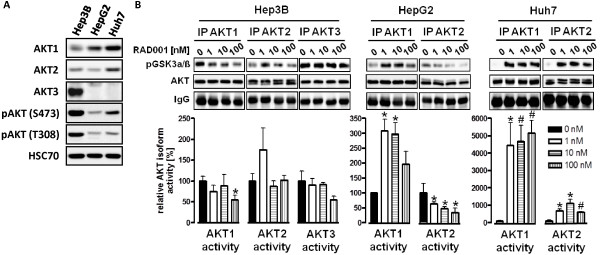
**Differential and isoform specific activation of AKT1, AKT2 and AKT3.** (**A**) Comparison of AKT isoform expression in HCC cell lines. Exponentially growing cells were seeded into 10 cm dishes and allowed to attach for 24 h. Cells were then lysed and analyzed by Western blot. One representative experiment out of three is shown. (**B**) HCC cells were treated over 24 h with the indicated concentration of RAD001. AKT isoform specific *in vitro* kinase assays were performed following quantitative AKT isoform immunoprecipitation, based on the same cell lysates as shown in Figure 1A and B. GSK3αbgr; fusion protein was used as an AKT substrate and phosphorylation at (S9/21) detected by Western blot. Columns, mean of three independent experiments; bars, SD. * p < 0,05; # p < 0,01.

The regulation of AKT kinase activity involves phosphorylation of threonine residue 308 in the activation loop and serine residue 473 in the hydrophobic region [32,33]. Of note, phospho-specific AKT antibodies directed against S473 and T308 used here and in numerous other publications do not discriminate between the different AKT isoforms. Therefore, the representation of single AKT isoform activities measured by the expression of pAKT (S473) or (T308) with phospho-AKT specific antibodies remains unclear. A more detailed insight into the regulation of AKT isoforms may be interesting in the term of developing of AKT isoform specific inhibitors as anticancer drugs. To assess whether a specific AKT isoform becomes predominantly activated after RAD001 treatment, we first analyzed the expression of AKT isoforms in HCC cell lines. As shown in Figure 2A, all cell lines expressed AKT1 and AKT2, with the highest expression levels observed in Huh7 cells (Figure 2A). In contrast, AKT3 expression was restricted to Hep3B cells.

To reveal changes in AKT activity, an AKT isoform-specific, quantitative *in vitro* kinase assay was performed as described previously [24], using the same cell lysates as shown in Figure 1A. Since AKT3 was not detectable in HepG2 and Huh7 cells either by western blot or by immunoprecipitation technique using different AKT3 antibodies, we analyzed AKT3 kinase activity only in Hep3B cells. Interestingly, we observed a differential concentration-dependent pattern of AKT-isoform activation following RAD001 treatment for the three HCC cell lines analyzed. Hep3B cells showed a moderate increase in AKT2 activity, but no increase in AKT1 and AKT3 activity (Figure 2B). The increase in AKT2 activity was only observed at 1nM RAD001 but not at higher concentrations. In contrast, in HepG2 cells we observed an increase in AKT1 activity at all concentrations analyzed, whereas AKT2 kinase activity was decreased in a concentration-dependent manner. In Huh7 cells, RAD001 led to a 40- and 20-fold increase in AKT1 and AKT2 kinase activity after stimulation with 1 nM RAD001, respectively. Interestingly, a similar activation of both AKT isoforms was also observed after stimulation of these cells with the higher concentration of 10 and 100 nM RAD001 (Figure 2B) [34].

### Dual targeting of mTOR and AKT highly synergistically inhibits proliferation of HCC cell lines

**Figure 3:**
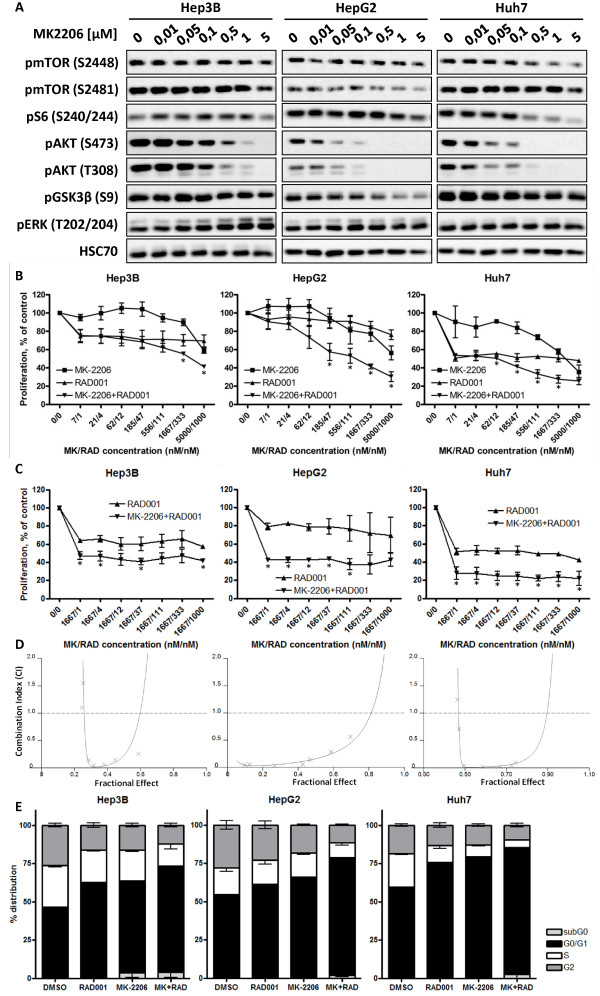
**Combining RAD001 with MK-2206 synergistically suppresses proliferation of HCC cell lines.** (**A**) HCC cell lines were treated with the indicated concentration of MK-2206 over 24 h, and changes in mTOR- and AKT-signaling were analyzed by Western Blot. HSC70 served as loading control. (**B**) HCC cells were seeded into 96 well plates and incubated with increasing concentrations of either RAD001 (triangle), MK2206 (box), or a combination (inverted triangle) of both with a fixed ratio of 1:5. Controls were treated with DMSO only. Proliferation was analyzed after 72 h BrdU-incorporation. Asterisks indicate a significantly stronger inhibition of the drug combination compared to each compound alone, * p < 0.05. (**C**) HCC cell lines were treated with RAD001 (triangle) or a combination of RAD001 and MK-2206 (inverted triangle) with a constant concentration of 1.7 μM MK2206 for 72 h. Cells treated with DMSO only served as control. Proliferation was analyzed as in (**B**). Each data point represents mean of at least three independent experiments, normalized to controls; bars, SD. Asterisks indicate a significantly stronger inhibition of the drug combination compared to each compound alone, * p < 0.05. (**D**) Fractional effect plot for the effect of RAD001 and MK2206 as seen in (**B**). (**E**) Cell cycle analysis of HCC cell lines after 24 h treatment with 100 nM RAD001, 1.7 μM MK-2206, or the combination of both, compared to DMSO treated controls. Colums: mean of one representative experiment, performed in triplicates; bars: SD. The drug combination resulted in a significant increase of cells in G0/G1 phase compared to each drug alone and compared to controls in all three cell lines (p < 0.05).

We next aimed to investigate the impact of AKT activity after RAD001 treatment. By use of MK-2206, a new highly potent allosteric pan-AKT inhibitor, we analyzed dual targeting of mTOR and AKT on proliferation of HCC cells. As shown in Figure 3A, AKT inhibitor MK-2206 reduced the phosphorylation of pAKT (S473) and (T308) in all HCC cell lines, with no preference for either phosphorylation site. Concomitant with the reduction in pAKT was a moderate reduction in phosphorylation of GSK3β at S9 and S6 at S235/246, albeit to a varying degree among the analyzed cell lines. To evaluate a potential synergistic effect of combined inhibition of mTOR and AKT, we used the method proposed by Chou and Talalay [35]. HCC cell lines were treated with RAD001, MK-2206, or a combination of both compounds with a fixed ratio of 1:5, over a broad range of clinically relevant concentrations. To exclude effects of plating density on proliferation, we established ideal plating densities for each cell line (Additional file 2: Figure S2). The importance of this step was underlined by a marked reduction of proliferation for cells reaching >80% confluence.

Treatment with RAD001 over 72 h resulted in a significant decrease in proliferation of all cell lines, with HepG2 cells being least, and Huh7 being most sensitive to RAD001 (Figure 3B).In contrast to Huh7 and Hep3B, HepG2 cells harbor a mutation in N-ras ( http://rcgdb.bioinf.uni-sb.de/MutomeWeb) which was discussed to contribute to RAD001 resistance [36]. We observed no significant dose dependent effects of RAD001 on proliferation for the concentrations between 1 nM and 1000 nM with the exception that HepG2 cells showed a non-significant trend towards stronger inhibition at higher concentrations of RAD001.

The anti-proliferative efficacy of AKT inhibitor MK-2206 alone was only weak with IC_50_ values of 3.7 μM, 7.4 μM and 3.1 μM for Hep3B, HepG2 and Huh7, respectively (Additional file 3: Figure S3). However, combining RAD001 and MK-2206 led to a synergistic suppression of proliferation in all three cell lines, with Combination Index (CI) values indicating strong, or very strong synergism especially at moderate and higher concentrations of the two compounds (Figure 3D, Additional file 4: Figure S4). As shown in Figure 3E combining RAD001 and MK-2206 resulted in a significantly higher accumulation of cells in G0/G1 phase compared to each compound alone. An increase of cells in subG1 phase was present in all cell lines treated with the combination, indicating the presence of apoptotic cells.

Furthermore, since effects of RAD001 alone on proliferation were not dose dependent, we next tested whether synergistic effects were dependent on higher doses of RAD001. We therefore treated HCC cells with increasing concentrations of RAD001 alone or in combination with a fixed dose of MK-2206. As shown in Figure 3C, strong synergistic effects of dual mTOR- and AKT-inhibition could already be achieved at doses of RAD001 as low as 1 nM. Interestingly, no RAD001 concentration-dependent differences in the synergistic effect of RAD001 and MK-2206 were observed, although low doses of RAD001,i.e. 1 nM, resulted in the strongest increase in phosphorylation of AKT at S473 (Figure 1A, B) and AKT isoform activity (Figure 2B).

### Knockdown of a single AKT isoform is synergistic with mTOR inhibition on proliferation of HCC cells

**Figure 4:**
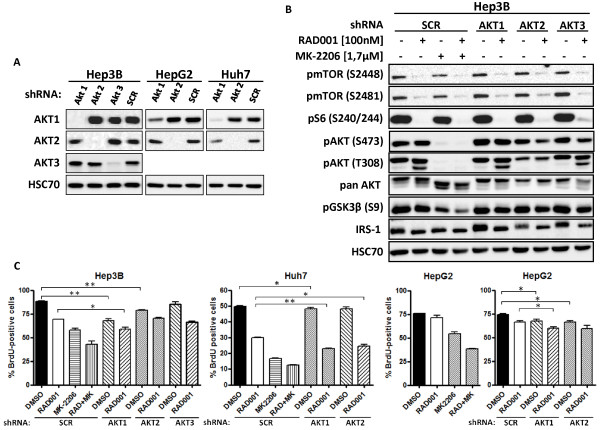
**Knockdown of AKT isoforms and mTOR inhibition synergistically inhibits HCC cell proliferation.** (**A**) shRNA mediated knockdown of single AKT isoform in HCC cell lines, confirmed by Western blot. (**B**) Hep3B knockdown cells were treated with 100 nM RAD001, 1.7 μM MK-2206, the combination of both, or DMSO as control, over 24 h, and mTOR and AKT signaling pathway activity was analyzed by Western blot. HSC70 served as loading control. AKT isoform knockdown cells were treated with 100nM RAD001 over 24 h. (**C**) Cells transduced with non-target vector were also treated with 1.7 μM MK-2206 or a combination of 100 nM RAD001 and 1.7 μM MK-2206. Proliferation was analyzed by BrdU incorporation. Columns, mean of one experiment performed in triplicates, bars, SD. *, p < 0.01; **, p < 0.001.

To analyze the impact of single AKT isoforms on proliferation of HCC cell lines, we generated stable AKT1 and AKT2 knockdown cells for all three HCC cell lines. Since we were unable to detect AKT3 at protein level in HepG2 and Huh7 cells, AKT3 knockdown cells were only established for Hep3B cells (Figure 4A). Knockdown of AKT isoforms was highly effective, and no significant changes in the expression of the remaining AKT isoforms were detected as shown for Hep3B cells.

Next we compared the effect of MK-2206 and knockdown of single AKT isoforms on activation of AKT and mTOR signaling pathways in HCC cell lines (Figure 4B for Hep3B cells, Additional file 5: Figure S5 for HepG2 and Huh7 cells). Treatment with 1.7 μM MK-2206 completely abolished phosphorylation of AKT and decreased phosphorylation of GSK3β at serine residue 9. Knockdown of AKT2 led to a reduced phosphorylation of AKT at S473and T308 in all three cell lines, whereas knockdown of AKT1 and AKT3 did not result in reduced levels of phosphorylated AKT (Figure 4B, Additional file 5: Figure S5).

To further investigate synergistic effects of combined mTOR and AKT isoform inhibition, AKT isoform knockdown cells were treated with 100 nM RAD001 or DMSO for 24 h. In addition, control cells treated with 1.7 μM MK-2206 alone or in combination with 100 nM of mTOR inhibitor RAD001 were analyzed. As shown in Figure 4C, knockdown of AKT1 led to a significant inhibition of proliferation of all investigated HCC cell lines, with the strongest effect observed in Hep3B cells. While depletion of AKT2 reduced proliferation of both, Hep3B and HepG2 cells, knockdown of AKT3 had no significant effect on proliferation of Hep3B cells. In combination with RAD001 knockdown of AKT1 resulted in a synergistic inhibition of proliferation of all HCC cell lines, whereas knockdown of AKT2 was only synergistic in Huh7 cells. Combining RAD001 with pan AKT inhibitor MK-2206 led to a markedly stronger reduction of cell proliferation than combining RAD001 with knockdown of any single AKT isoform.

### RAD001 and MK-2206 are highly synergistic *in vivo*

**Figure 5:**
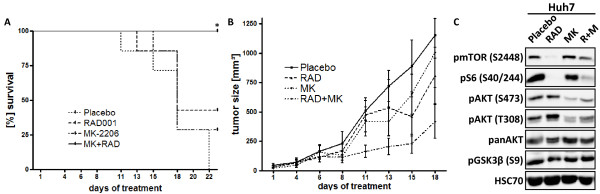
**RAD001 and MK-2206 synergistically suppress subcutaneous tumor growth in vivo.** Huh7 cells were injected subcutaneously into SCID mice (n = 7 per group). After formation of palpable tumors, mice were treated with Placebo, RAD001, MK-2206 or the combination of both, respectively. (**A**) Treatment with RAD001 and MK-2206 significantly prolonged survival of tumor bearing mice compared to all other groups (p < 0.05). (**B**) Tumor volume was monitored over an 18 day period, presented as mean ± SEM. RAD + MK vs Placebo and RAD + MK vs MK-2206 alone was significant at day 15 (p < 0.05). Note that one mouse had to be withdrawn from the experiment in the RAD001 and MK-2206 treatment group at day 13 and 15 respectively, and two mice were withdrawn from the Placebo group at days 11 and 15. (**C**) AKT and mTOR signaling in tumor samples was analyzed by Western blot.

Next, we analyzed the efficacy of dual treatment with RAD001 and MK-2206 in a subcutaneous HCC xenograft mouse model. As seen in Figure 5A, combining RAD001 and MK-2206 significantly prolonged survival of mice compared to placebo or each compound alone. Furthermore, a significant decrease in tumor volume compared to placebo was only observed in mice treated with the combination of RAD001 and MK-2206 (Figure 5B). Until 15 days post-treatment, no significant difference in mouse weight was observed between the four groups, indicating that the treatment was well tolerated. The decrease in the average weight at day 18 in placebo and MK-2206 treated mice reflects tumor cachexia due to tumor progression (Additional file 6: Figure S6). To confirm specific effects of RAD001 and MK-2206 on PI3K/AKT/mTOR signaling, tumor portions from one randomly chosen mouse per group were subjected to Western blot analysis. As seen in Figure 5C, RAD001 almost completely abolished phosphorylation of S6 at serine residue 240/244. Treatment with RAD001 slightly increased the phosphorylation of AKT at threonine residue 308, whereas treatment with MK-2206 suppressed the phosphorylation of AKT at both serine residue 473 and threonine residue 308.

### All AKT isoforms are activated in a HCC patient with mutated PI3K

In order to identify genetic alterations in the PI3K/AKT/mTOR signaling pathway in liver cancer, genomic DNA from 10 primary tumor samples of HCC patients were analysed by direct sequencing after PCR amplification. Yet, mutations in the regulatory p85α subunit of PI3K, the PH domain of the three AKT isoforms and specific domains of mTOR have not been analysed in HCC. The 47 exons of the sequenced genes PIK3R1 (p85α subunit of PI3K), PIK3CA (catalytic subunit of PI3K) FRAP1 (mTOR), the three AKT isoforms (AKT1, AKT2, AKT3) and the genetic alterations identified within the 10 HCC samples are summarized in Table 1. No genetic alterations have been identified in *AKT1, AKT2* or *AKT3.* A *c*omparison with the NCBI SNP database revealed that genetic alterations detected in PIK3R1 and mTOR (FRAP1) are SNPs. One somatic mutation was identified in PIK3CA encoding the catalytic subunit of PI3K in tumor sample H292 (exon 20, 3140 a > g; H1047R).

**Table 1:** Mutation analysis of genes encoding PI3K, mTOR and AKT isoforms in primary HCC samples

**Patient ID**	**PI3KR1**	**PI3KCA**	**mTOR**	**AKT1**	**AKT2**	**AKT3**
**H282**			6909 g > ga			
**H358**	978 g > ga					
**H43**			6909 g > ga			
**H146**						
**H219**	978 g > ga					
**H279**	978 g > ga		6909 g > ga			
**H292**	1176 c > ct	3140 a > g	6909 g > ga			
**H361**			6909 g > ga			
**H59**			6909 g > ga			
**H326**						

**Figure 6:**
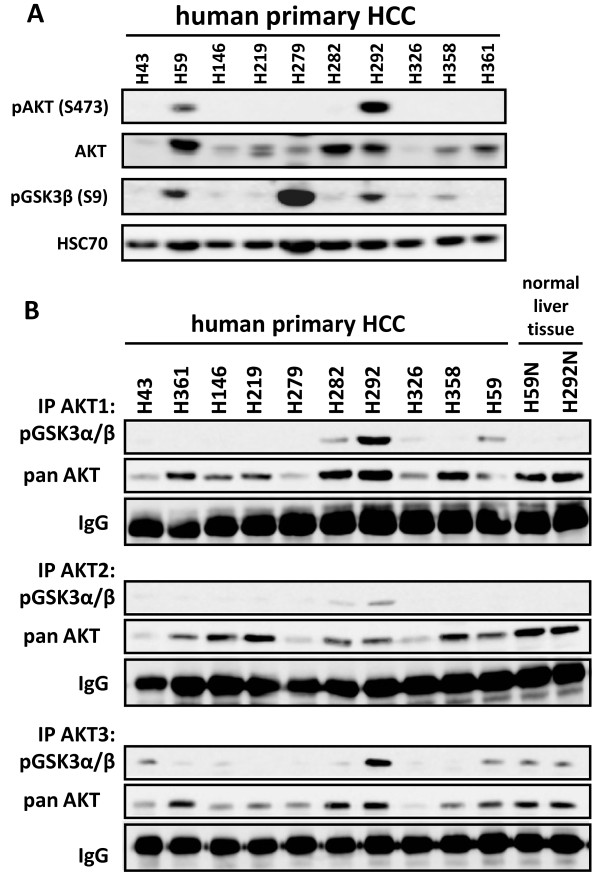
**AKT isoforms are differentially activated in human HCC tissue samples.** (**A**) Ten human HCC tissue samples obtained from specimen removed during surgery were analyzed by Western blot for expression of mTOR and AKT signaling proteins. HSC70 served as loading control. (**B**) Same samples, including two corresponding controls from surrounding normal liver tissue, were analyzed by AKT isoform specific *in vitro* kinase assay as described before. IgG served as loading control.

 To further gain insight into the state of AKT activation and signaling in HCC *in vivo*, tumor samples used for sequencing have additionally been analysed on protein level by western blotting (Figure 6A). As expected, we observed heterogeneous expression profiles of phosphorylated AKT. AKT was activated in two out of ten investigated HCC samples, and functional AKT signaling in these two tumor samples was further confirmed by a concomitant phosphorylation of the AKT downstream ubstrate GSK3β (S9).

The activation status of AKT isoforms was further analysed by AKT isoform specific *in vitro* kinase assays. As shown in Figure 6B, AKT isoform expression does not correlate with AKT isoform activity, indicating that AKT isoforms are differentially activated independent of their expression level *in vivo*. Furthermore, we demonstrated for the first time that the H1047R mutant in the catalytic subunit of PI3K led to elevated activity of all AKT isoforms in comparison to healthy liver tissue of the patient (see patient H292, Table 1). These results indicate that all AKT isoforms become activated by the oncogenic hot spot H1047R mutant of PI3K in HCC.

## Discussion

In this study, we aimed to investigate the PI3K/AKT/mTOR signaling pathway in hepatocellular carcinoma by highlighting the feedback activation of AKT and its distinct isoforms following mTOR inhibition by RAD001. Further, we analyzed the activation status of specific AKT isoforms in HCC samples and corresponding healthy liver tissue as well as their diverse implications in terms of proliferation of HCC cell lines. In order to draw a light on genetic alterations of PI3K/AKT/mTOR signaling in hepatocellular carcinoma, DNA sequencing of human tumor samples were performed. The molecular events leading to activation of the PI3K/AKT/mTOR signaling cascade are not fully understood. In general, mutations in mTOR are rare. Two single amino acid substitutions in TOR1 (FRB domain) have been identified in yeast, exhibiting resistance to caffeine [37]. In addition, a point mutation in TOR2 (FRB domain) was found leading to a weak rapamycin resistance [38]. On humans, two oncogenic mutations of mTOR have been reported so far in colorectal and renal carcinoma [39,40]. Genetic aberrations in AKT itself have not yet been identified in HCC patients. In contrast, mutations in *PIK3CA*, the gene encoding for the catalytic subunit of PI3K, have been described in up to 35% of patients with HCC [41-43]. Our mutational analysis of ten human HCCs revealed no genetic alteration in mTOR or in any of the AKT isoforms. One somatic “hot spot” mutation of PI3K was detected in the gene *PIK3CA* (exon 20, 3140 a > g [H1047R]). Moreover, we analyzed the expression and activity of AKT isoforms in these tumor samples. Although all AKT isoforms were expressed in HCC samples, results revealed that AKT activity is often confined to particular AKT isoforms and independent of AKT isoform expression. In this study, we demonstrate for the first time that the oncogenic and well characterized H1047R mutant of PIK3CA, leading to constitutive activation of PI3K [44], results in elevated activity of all three AKT isoforms in comparison to healthy liver tissue of this patient. These data indicate that all AKT isoforms become activated by mutant PI3K (H1047R) in HCC. Whether all AKT isoforms are involved in the oncogenic signalling has to be shown in further experiments.

Our data further demonstrate that combining mTOR inhibitor RAD001 and AKT inhibitor MK-2206 shows a strong synergistic effect on the proliferation of HCC cell *in vitro* and *in vivo*. Therefore, dual targeting of AKT and mTOR might be a promising treatment option for HCC patients. Moreover, we give new insight into AKT isoform specific kinase activity and signaling after mTOR inhibition in HCC cells. Increased AKT1 kinase activity following mTOR treatment was reported before in HeLa cells [30]. However, AKT2 and AKT3 kinase activity has not been investigated. Our data demonstrate that single AKT isoforms can be differentially regulated after mTOR inhibition, as shown for HepG2 cells. Therefore, analysis of all AKT isoforms is necessary to understand the complex mechanism of AKT signaling. Overall, we demonstrated that a high expression level of phosphorylated AKT induced by feedback activation following mTOR inhibition can actually represent increased kinase activity of different AKT isoforms. The clinical relevance of this finding is underscored by the fact that increased AKT phosphorylation was recently observed in a significant proportion of tumor samples obtained from patients treated with RAD001 [45]. Which AKT isoforms become activated in patients treated with RAD001 has to be analyzed in further experiments.

In this study, we showed that knockdown of a single AKT isoform had only a minor effect on proliferation of HCC cells, with the most dominant effect observed for AKT1 in Hep3B cells. Although specific functions of AKT isoforms have clearly been demonstrated [26,46], compensation and redundancy among AKT isoforms might limit the efficacy of AKT isoform specific inhibitors. However, further research is necessary to understand the exact effect of single AKT isoform inhibition on function and activity of remaining AKT isoforms.

Functional analysis of AKT isoforms of different tumor entities revealed specific oncogenic functions for distinct AKT isoforms [47]. Conclusively, the group of Muller demonstrated that AKT1 induced tumor growth, while AKT2 promotes metastasis of ErbB2-induced breast cancer *in vivo*[48]. Taking together these results suggest that cancer patients would profit more of a pan AKT inhibitor than of AKT isoform specific inhibitors. Consequently, inhibition of mTOR by RAD001 in combination with knockdown of a single AKT isoform evoked only small synergistic effects on proliferation of HCC cells and was additionally not restricted to a single AKT isoform as shown for Huh7 cells. In order to investigate dual targeting of mTOR and total AKT, we analyzed the efficacy of combination of mTOR inhibitor RAD001 with the potent pan AKT inhibitor MK-2206. In contrast to single AKT isoform knockdowns, treatment with pan AKT inhibitor MK-2206 in combination with RAD001 led to a significant, highly synergistic reduction of cell proliferation of all HCC cell lines tested. These data suggest that the inhibition of all AKT isoforms using a pan AKT inhibitor like MK-2206 is necessary to achieve profound synergistic effects in combination with the mTOR inhibitor RAD001. Highlighting the dual targeting of mTOR and AKT as an efficient therapy approach of HCC is of great clinical interest, since MK-2206 is already being tested in clinical trials and reported to be well tolerated [49,50].

## Conclusion

In summary, our data demonstrate that dual targeting of mTOR and AKT led to synergistic inhibition of proliferation of HCC cell lines. Further, our results suggest that inhibition of all, rather than one specific AKT isoform is necessary to achieve bold synergistic effects on cell proliferation. In conclusion, the combination of mTOR inhibitor RAD001 and pan AKT inhibitor MK-2206 might represent a new promising treatment strategy for patients with advanced HCC.

## Methods

### Materials

RAD001 was provided by Novartis (Basel, Switzerland). MK-2206 was obtained from Selleck Chemicals (Houston, TX, USA). Stock solutions with a concentration of 10 mM were prepared and stored at −80°C. All RAD001 solutions were thawed and refrozen for a maximum of three times and then discarded. Antibodies against pan AKT, AKT1, AKT2, pAKT (S473), pAKT (T308), mTOR, pmTOR (S2448), pmTOR (S2481), Raptor, Rictor, pERK (T202/204), ERK, pS6 (S240/244), IRS-1 and pIRS-1 (S636/639) were purchased from Cell Signaling Technology (Danvers, MA). Antibodies against AKT2 and HSC-70 were purchased from Santa Cruz. AKT3 antibody was obtained from Millipore (Schwallbach, Germany). 7-AAD was obtained from BD Biosciences (Pharmingen, CA, USA).

### Cell culture

The three hepatocellular carcinoma cell lines Hep3B, HepG2 and Huh-7 were a kind gift from Prof. Dr. H. Will at the Heinrich Pette Institute, Hamburg, Germany. All cell lines were maintained in DMEM, supplemented with 10% (v/v) FCS, and 1% (v/v) penicillin and streptomycin. Cells were cultured at 37°C in a humidified atmosphere containing 5% CO_2_. All cells were tested for mycoplasma contamination every 2–3 months.

### Western Blot analysis

Western blot analysis was performed as described previously [24]. Protein expression was quantified using an LAS-3000 Imager from Fuji (Raytest, Straubenhardt, Germany).

### Lentiviral knockdown of AKT isoforms

pLKO.1-puro vector encoding either AKT1, AKT2, AKT3 or scrambled shRNA were purchased from Sigma-Aldrich (Taufkirchen, Germany). Generation of pseudotype lentiviruses and transduction were performed as previously described [24]. Transduced cells were selected by addition of puromycin (Sigma-Aldrich, Taufkirchen, Germany) to culture medium (final concentration 1.5 μg/ml) for at least two weeks before experiments were carried out.

### Proliferation and cell cycle assay

Proliferation was analyzed either by flow cytometry using the BrdU APC Flow Kit (BD, Pharmingen, CA, USA) or with the colorimetric BrdU ELISA Kit (Roche®, Basel, CH) as indicated in the figure legends. For FACS-based assays, cells were seeded into 10 cm dishes and allowed to attach overnight. Then, medium was replaced by medium containing RAD001, MK-2206, a combination of both, or DMSO as control. Final DMSO concentration in culture medium was 0.1% (v/v) in all experiments. For labeling BrdU (final concentration 10 μM) was added and cells were incubated for 12 to 16 h. For cell cycle analysis, cells were fixed in ice cold 70% ethanol for at least 6 h, washed and subsequently incubated with 5 μg 7-AAD and 5 μg RNAse A for one hour. Each experiment was performed in triplicates and has been repeated at least one time. Analysis was performed on BD Canto II flow cytometer (BD Pharmingen, CA, USA). Cell cycle analysis was performed using FlowJo 7.6.5 software.

For BrdU ELISA assays, cells were seeded into 96-well plates and allowed to attach overnight. Cells were then incubated for 72 h with different concentrations of MK-2206, RAD001, or a combination of both. Controls were treated with DMSO only. BrdU ELISA was performed as described by the manufacturer (Becton Dickenson, Heidelberg, Germany). Each experiment was repeated at least three times in quadruplicates.

### Immunoprecipitation and AKT isoform specific *in vitro* kinase assay

Immunoprecipitation of AKT isoforms and subsequent *in vitro* kinase assay was performed as described before [24]. Whole samples were analyzed by western blot technique probed with pGSK3α/ß (S9/21) and panAKT antibody. Subsequently,nitrocellulose membrane was incubated with secondary goat anti-mouse antibody (Santa Cruz Biotechnology, CA, USA) to detect mouse IgG levels for sample correction.

### Primary human HCC samples

The human investigations were performed according to the Declaration of Helsinki after approval was obtained by the local ethics committee of the Medical Association Hamburg. From all patients, written informed consent was obtained prior to study related procedures. Tumor samples and samples of corresponding healthy liver tissue of HCC patients treated at the University Medical Center Hamburg Eppendorf, Department of Hepatobiliary and Transplant Surgery were stored at Indivumed (Hamburg, Germany) following the Indivumed Standard of Biobanking ( http://www.indivumed.com). Genomic DNA isolation and sequence analysis was performed by Inostics (Hamburg, Germany). For protein analysis and kinase assays tissue samples were lysed by homogenisation of samples with Lysis Matrix-D (MP, USA) in NP-40 lysis buffer (containing: 50 mM HEPES pH 7.5, 150 mM NaCl, 1% NP-40, 2% aprotinin, 2 mM EDTA, 50 mM NaF, 10 mM NaPPi, 10% glycin, 1 mM vanadate and 1 mM PMSF) with the tissue lyser MX Pro (MP, USA).

### Mutation analysis of PI3K, AKT and mTOR in HCC samples

Sequence analysis of genes from 10 HCC samples encoding the p85α adapter subunit of PI3K (exon 9–17 of PIK3RI), the p110α catalytic subunit of PI3Kα (exon 2 and 10–21 of PIK3CA), mTOR (exon 44–57 of FRAP1), AKT1 (exon 3–6), AKT2 (exon 2–5) or AKT3 (exon 1–3) were performed by amplification of the exons with primers located in the neighboring introns by PCR and sequenced using BigDye Terminator 3.1 according to the instructions by the manufacturer (Applied Biosystems). Primer sequences can be found in Additional file 7: Table S1.

### Animal experiments

All experimental protocols were approved by local authorities (Ministry of Health and Consumer Protection, Hamburg, Germany, Permit Number G52/11). 2x10^6 Huh7 cells were injected subcutaneously into SCID mice (female, age 8 weeks, n = 7 per group, obtained from Charles River, Sulzfeld, Germany). Upon establishment of palpable tumours after three weeks, mice were randomly assigned to one of the four groups (Placebo, RAD001, MK-2206, or RAD001 and MK-2206), and treatment was started. Tumor growth was monitored by regular visual inspection and tumor dimensions were measured every 2–3 days. Tumor volume was calculated using the formula longest tumor diameter x (shortest tumor diameter)^2/2. RAD001, formulated as a microemulsion, was dosed at 1 mg/kg body weight and administered daily Monday through Friday. MK-2206 was formulated in a 30% (w/v) Captisol solution and administered Monday, Wednesday and Friday dosed at 100 mg/kg. A placebo microemulsion (provided by Novartis) and a 30% (w/v) Captisol solution served as placebo. The compounds were mixed immediately before administered by gavage in a total volume of 100 μl. Animals were treated until termination criteria (tumor ulceration, tumor size > 2 cm in largest diameter, loss of body weight exceeding 20%) were met, or for a maximum of 22 days when all mice in the placebo group had to be withdrawn. Xenograft primary tumours were harvested at necropsy and a portion of each tumor was snap frozen on liquid nitrogen for Western Blot analysis.

### Statistical analysis

Student’s *t*-Test (unpaired, 2-tailed) was calculated based on the data of at least three independent experiments. Bonferroni correction for multiple testing was performed where applicable. Results were considered significant if p < 0.05. All error bars represent SD. Drug interactions were analyzed based on the median effect method of Chou and Talalay [35]. CalcuSyn software (Biosoft, Cambridge, UK) was used to calculate the combination index (CI). CI values from 0.3 to 0.7 are considered to indicate synergism, CI values below 0.3 are considered to represent strong synergism, and values below 0.1 very strong synergism. IC_50_ values were calculated using CurveExpert Professional 1.3 software. Chi-squared test was used to test for significant differences in mouse survival.

## Abbreviations

mTOR: Mammalian target of rapamycin; PI3K: Phosphatidylinositol 3-kinase; PI3KCA: Catalytic subunit of phosphatidylinositol 3-kinase; IRS-1: Insulin receptor substrate 1; BrdU: 5-bromo-2′-deoxyuridine; DMSO: Dimethyl sulfoxide; PBS: Phosphate buffered saline; HCC: Hepatocellular carcinoma; CI: Combination index.

## Competing interests

The authors declare that they have no competing interests.

## Authors’ contributions

NG, FE, BH, KS, US, BN and MJ designed the study. FE, NG and MJ performed the experiments and interpreted the experimental findings. FE and NG drafted the manuscript. FE, NG and MJ wrote the final version of the manuscript. All authors read and approved the final manuscript.

## Supplementary Material

Additional file 1 **Figure S1.** No increase in AKT phosphorylated at T308 or S473 is detectable in Hep3B cells after RAD001 treatment. Hep3B cells were treated with 100 nM RAD001 up to 72 h, and cell lysates were prepared at the indicated time points. Where indicated, medium was removed after 48 h and replaced by fresh, 100 nM RAD001 containing medium. Cell lysates were analyzed for AKT and mTOR signaling. HSC70 was used as loading control. Click here for file

Additional file 2 **Figure S2.** Influence of plating density on proliferation of HCC cell lines. Increasing numbers of HCC cells were seeded into 96-wells and incubated with different concentrations of RAD001 for 72 h. Proliferation was subsequently analyzed by BrdU incorporation. One representative experiment out of two is shown. Click here for file

Additional file 3 **Figure S3.** Determination of IC_50_ for MK-2206 in HCC cell lines. HCC cells were seeded into 96 well plates and incubated with increasing concentrations of MK-2206, controls were treated with DMSO only. Proliferation was analyzed after 72 h by detection of BrdU incorporation. Columns: mean of three independent experiments; bars: SD. Click here for file

Additional file 4 **Figure S4.** Effect of RAD001 alone or in combination with MK-2206 on HCC cell proliferation. HCC cells (2,5E5 cells for Hep3B and HepG2, 2E5 cell for Huh7) were treated with DMSO, 100 nM RAD001, 1.7 μM MK-2206, or the combination of both. The numbers of viable cells were counted using a Neubauer counting chamber and Trypane blue exclusion after 24, 48 and 72 h treatment. The combination of both compounds inhibits cell proliferation significantly stronger than placebo or each drug alone. * p < 0.05. Click here for file

Additional file 5 **Figure S5.** Effect of single AKT isoform knockdown of AKT and mTOR signaling. HepG2 and Huh7 AKT isoform knockdown cells were treated with 100 nM RAD001, 1.7 μM MK-2206, the combination of both, or DMSO, over 24 h, and mTOR and AKT signaling pathway activity was analyzed by Western blot. HSC70 served as loading control. Click here for file

Additional file 6 **Figure S6.** Treatment of mice bearing subcutaneous HCC-tumors had no effect on body weight. Mice treated with Placebo, RAD001, MK-2206 or both compounds in combination were weighed every other day during the first 18 day treatment period. Until day 15, no statistically significant changes in body weight were detected. Weight loss at day 18 in MK-2206 and Placebo treated animals was due to tumor cachexia, and these animals had to be withdrawn from the experiment. Data are presented as mean ± SEM. Click here for file

Additional file 7 **Table S1.** Sequencing primers used for mutation analysis. Click here for file
