# Supplementary figures and images for: Combined targeting of AKT and mTOR synergistically inhibits proliferation of hepatocellular carcinoma cells
Source: Mol Cancer. 2012 Nov 20;11:85. doi: 10.1186/1476-4598-11-85 (PMC3545733; doi:10.1186/1476-4598-11-85)

# Fig. S1

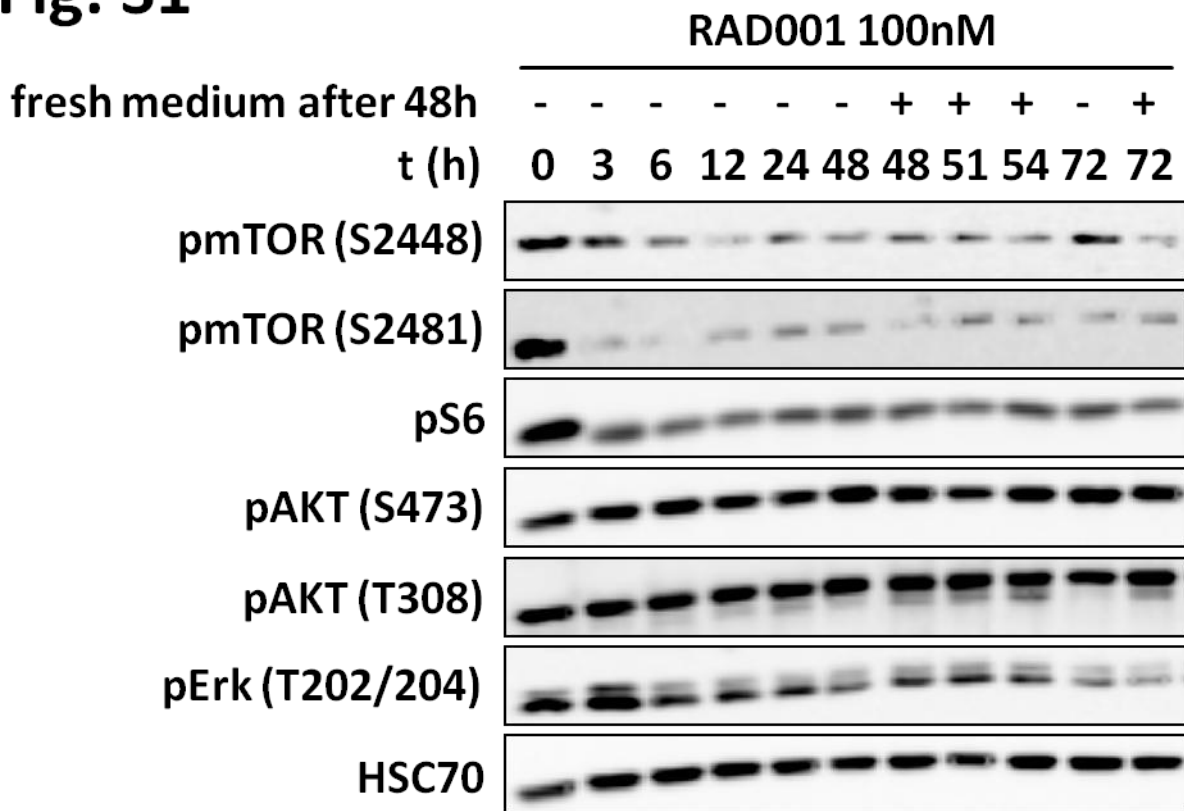

Supplement: Additional file 1 — Figure S1. No increase in AKT phosphorylated at T308 or S473 is detectable in Hep3B cells after RAD001 treatment. Hep3B cells were treated with 100 nM RAD001 up to 72 h, and cell lysates were prepared at the indicated time points. Where indicated, medium was removed after 48 h and replaced by fresh, 100 nM RAD001 containing medium. Cell lysates were analyzed for AKT and mTOR signaling. HSC70 was used as loading control. [file 1476-4598-11-85-S1.pdf]

**Fig. S2**

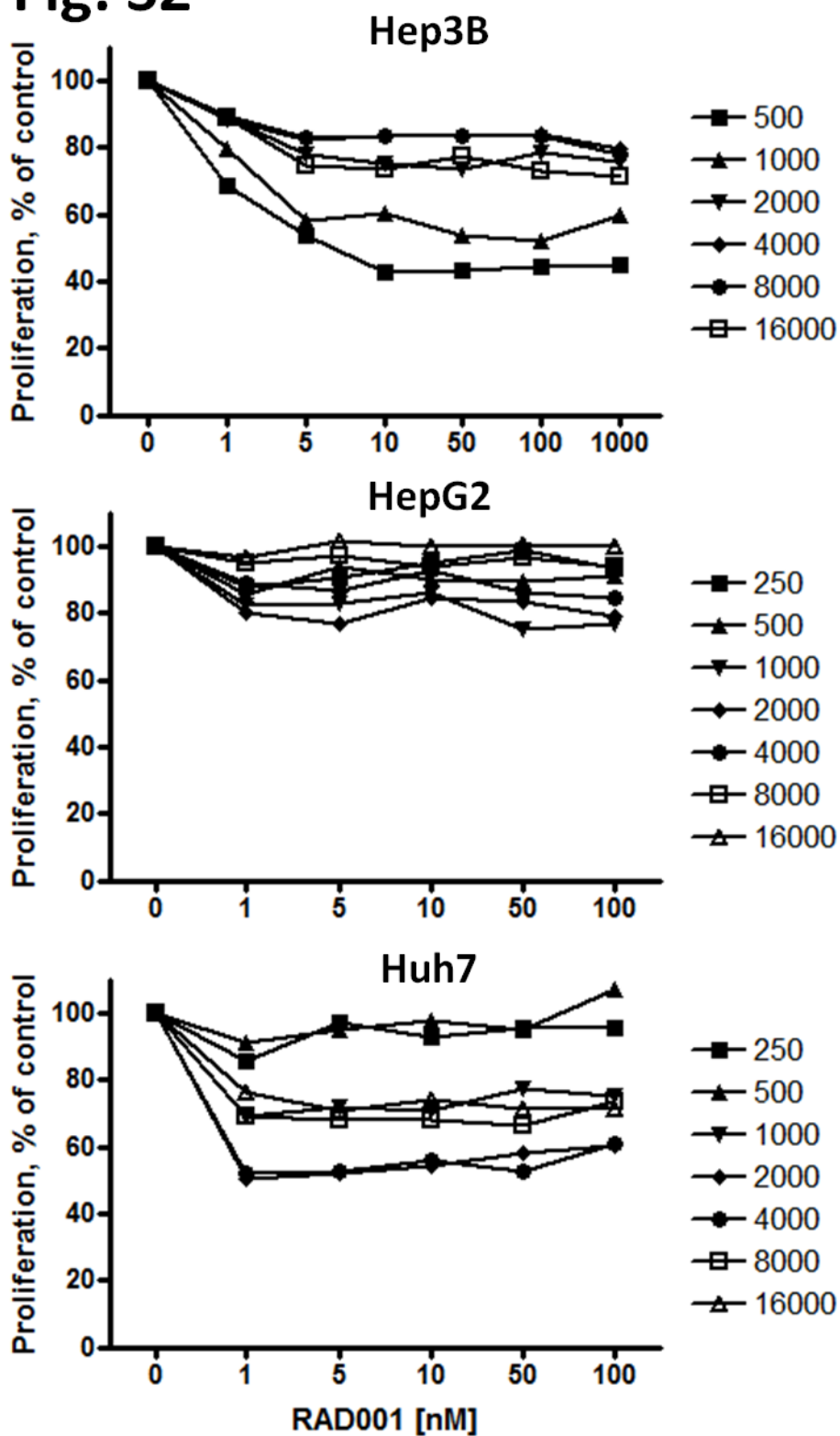

Supplement: Additional file 2 — Figure S2. Influence of plating density on proliferation of HCC cell lines. Increasing numbers of HCC cells were seeded into 96-wells and incubated with different concentrations of RAD001 for 72 h. Proliferation was subsequently analyzed by BrdU incorporation. One representative experiment out of two is shown. [file 1476-4598-11-85-S2.pdf]

**Fig. S3**

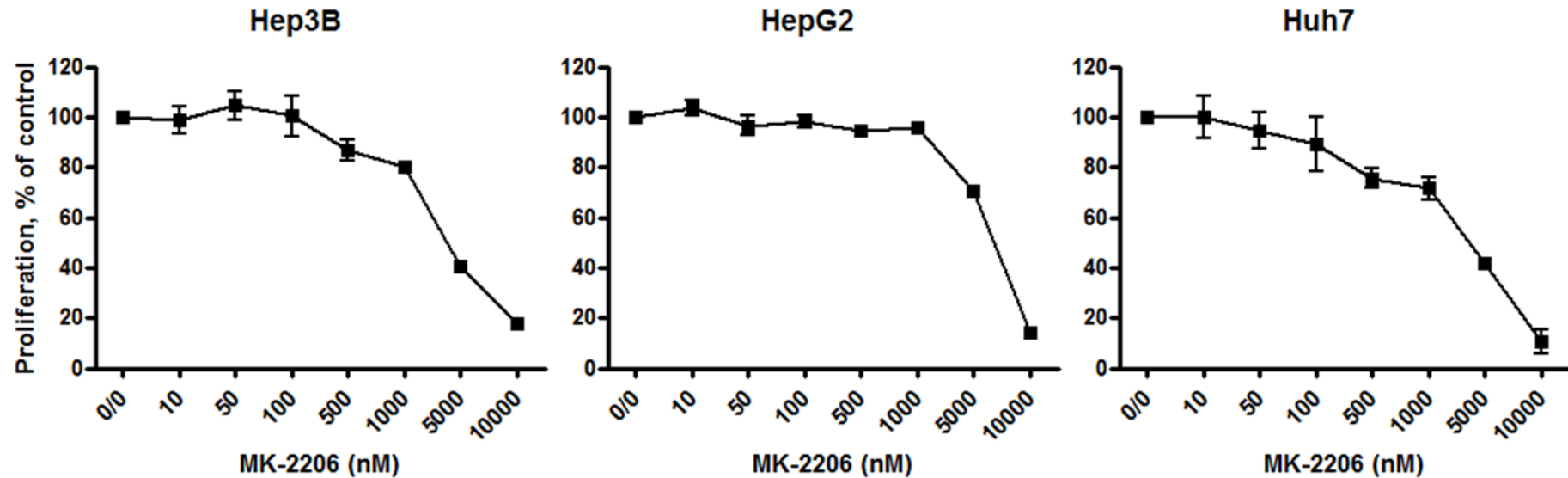

Supplement: Additional file 3 — Figure S3. Determination of IC50 for MK-2206 in HCC cell lines. HCC cells were seeded into 96 well plates and incubated with increasing concentrations of MK-2206, controls were treated with DMSO only. Proliferation was analyzed after 72 h by detection of BrdU incorporation. Columns: mean of three independent experiments; bars: SD. [file 1476-4598-11-85-S3.pdf]

**Fig. S4**

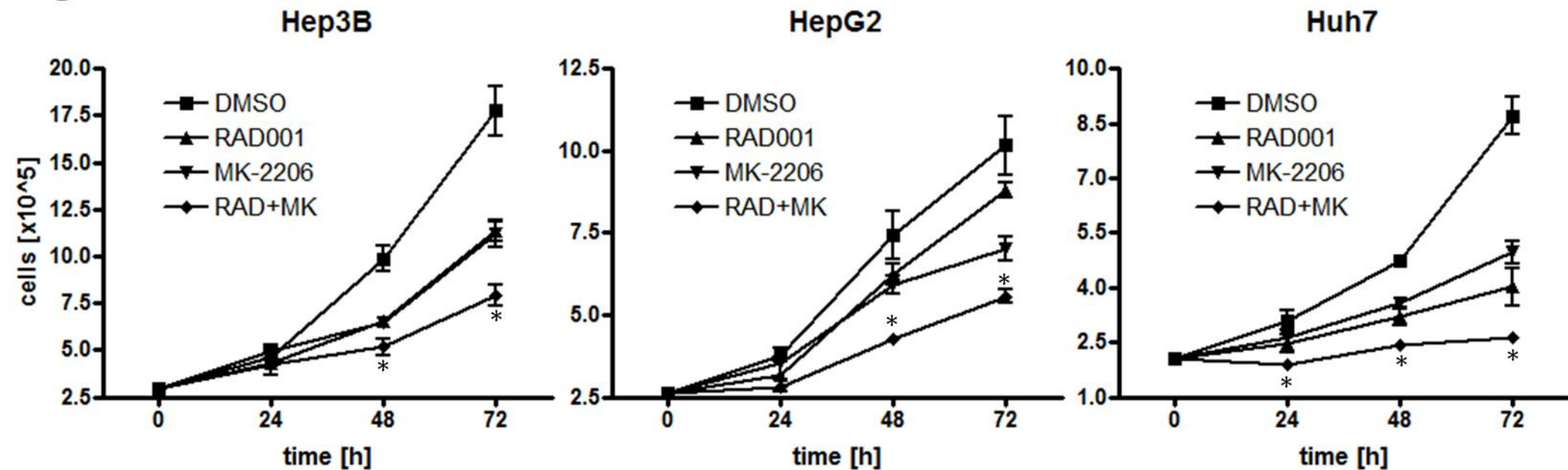

Supplement: Additional file 4 — Figure S4. Effect of RAD001 alone or in combination with MK-2206 on HCC cell proliferation. HCC cells (2,5E5 cells for Hep3B and HepG2, 2E5 cell for Huh7) were treated with DMSO, 100 nM RAD001, 1.7 μM MK-2206, or the combination of both. The numbers of viable cells were counted using a Neubauer counting chamber and Trypane blue exclusion after 24, 48 and 72 h treatment. The combination of both compounds inhibits cell proliferation significantly stronger than placebo or each drug alone. * p < 0.05. [file 1476-4598-11-85-S4.pdf]

**Fig. S5**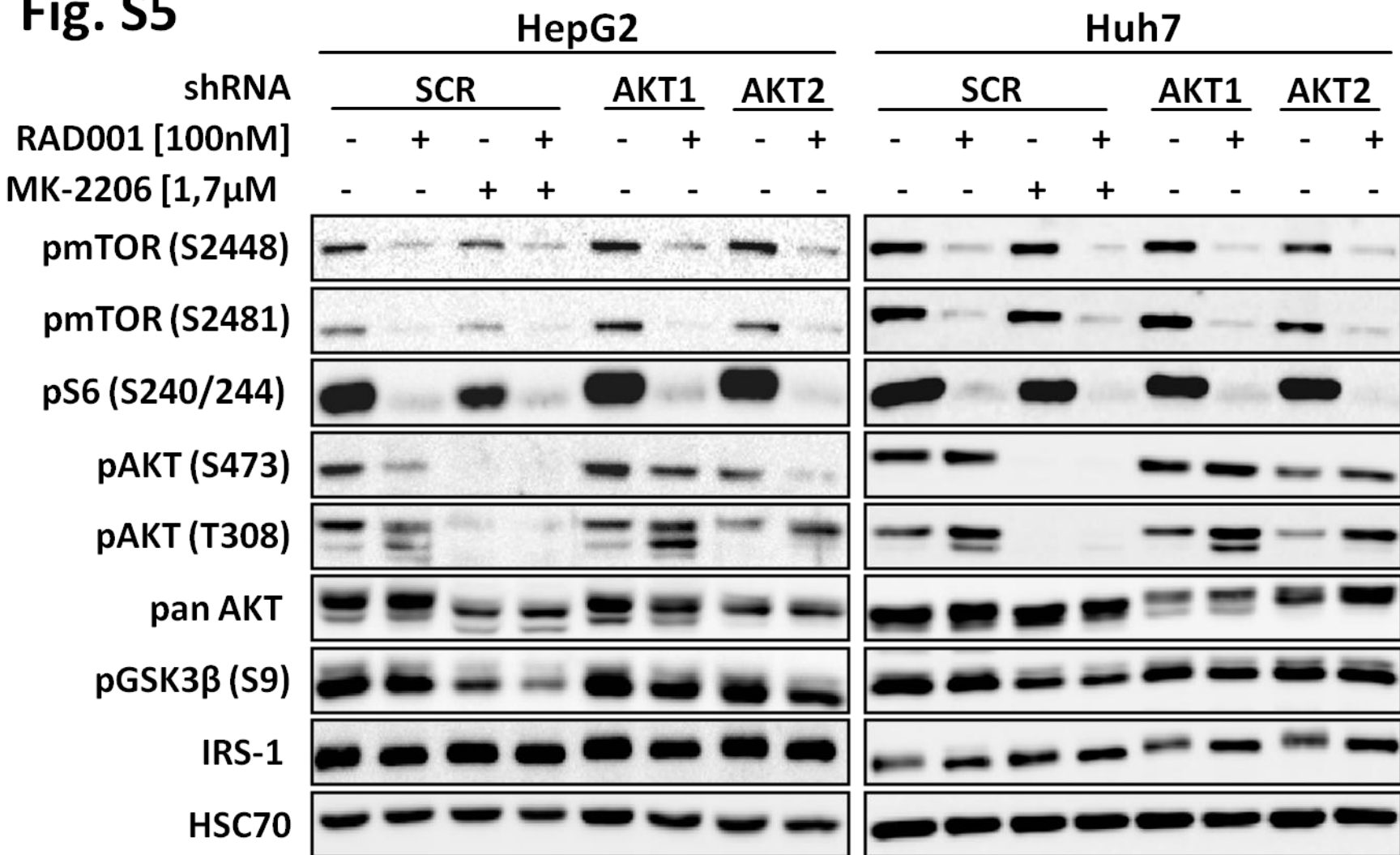

Supplement: Additional file 5 — Figure S5. Effect of single AKT isoform knockdown of AKT and mTOR signaling. HepG2 and Huh7 AKT isoform knockdown cells were treated with 100 nM RAD001, 1.7 μM MK-2206, the combination of both, or DMSO, over 24 h, and mTOR and AKT signaling pathway activity was analyzed by Western blot. HSC70 served as loading control. [file 1476-4598-11-85-S5.pdf]

**Fig. S6**

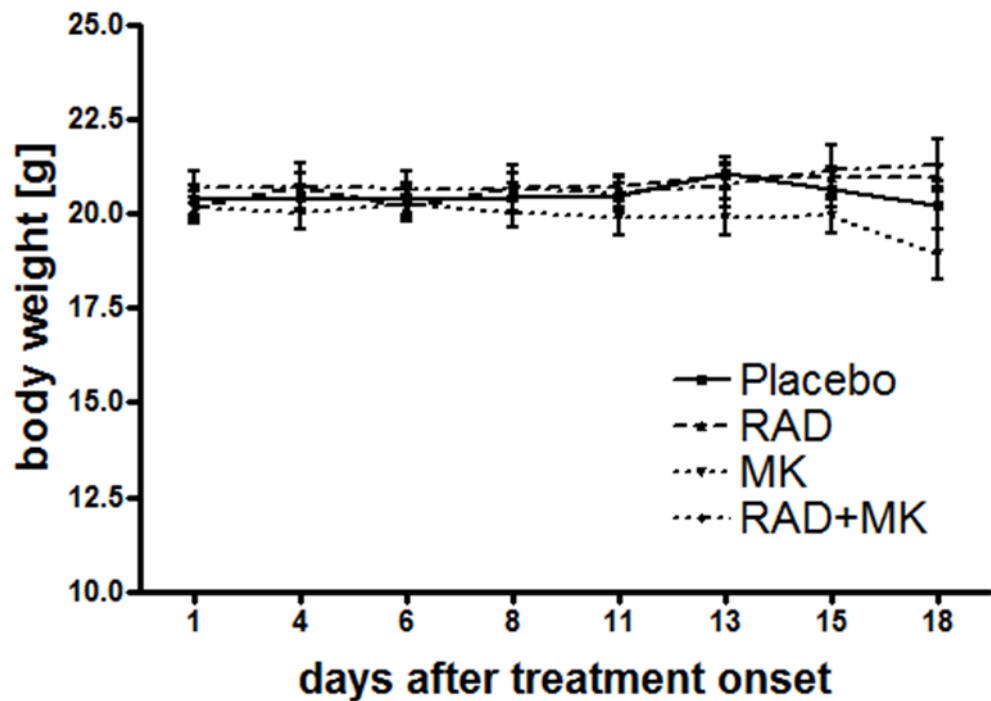

Supplement: Additional file 6 — Figure S6. Treatment of mice bearing subcutaneous HCC-tumors had no effect on body weight. Mice treated with Placebo, RAD001, MK-2206 or both compounds in combination were weighed every other day during the first 18 day treatment period. Until day 15, no statistically significant changes in body weight were detected. Weight loss at day 18 in MK-2206 and Placebo treated animals was due to tumor cachexia, and these animals had to be withdrawn from the experiment. Data are presented as mean ± SEM. [file 1476-4598-11-85-S6.pdf]
