# Supplementary material for: Combined targeting of AKT and mTOR synergistically inhibits proliferation of hepatocellular carcinoma cells
Source: Mol Cancer. 2012 Nov 20;11:85. doi: 10.1186/1476-4598-11-85 (PMC3545733; doi:10.1186/1476-4598-11-85)
Supplement: Additional file 7 — Table S1. Sequencing primers used for mutation analysis. [file 1476-4598-11-85-S7.doc]

***Table S1: Sequencing primers used for mutation analysis***

| **Gene** | **Sequence**  **Primer Forward** | **Sequence**  **Primer Reverse** |
| --- | --- | --- |
| PIK3R1Ex9 | CCATATTGCATGGAATTGTGAAC | AATGGAACTTAGCAAGCTGGTG |
| PIK3R1Ex10 | TTAAATCTATGTGGGCAGGAGG | TTTCACATCCAATTTGGGATTA |
| PIK3R1Ex11 | GGTAAGCCAGGGAATATAGCTG | TGCAATTATGTAATTACGAGTGTCAAG |
| PIK3R1Ex12 | TTAAAGATGTTTCCATGTCAGCTATT | TTGAAACTCAGTGACTGCTTCAG |
| PIK3R1Ex13 | GCAGTAAGAGATTGTTCTATGAAAGG | TCCACGTGATCATTCAAAGC |
| PIK3R1Ex14 | ATGTTGAGCCACTCCAAAAA | CCCAACCACTCGTTCAACTT |
| PIK3R1Ex15 | TCCAGCTGAGAAAGACGAGAG | TTCAGGGACATCATTATGGACAC |
| PIK3R1Ex16 | GGTATGCCTAGGGAAGACAGC | GGAATCCAAACCTAGTCTTCCAAC |
| PIK3R1Ex17 | CCCAAGTTGAGACTGCACAA | GCCTTAGGCTGCATGTCTTC |
| PIK3CAEx2a | GCCTAATCAAGTCAAACTATGGAAA | GAAAAAGCCGAAGGTCACAA |
| PIK3CAEx2b | CACGACCATCATCAGGTGAA | ACGAAGGTATTGGTTTAGACAGA |
| PIK3CAEx10 | CTGTGAATCCAGAGGGGAAA | GCATTTAATGTGCCAACTACCA |
| PIK3CAEx11 | AAAGCTAGTAATGTAAGAAGTTTGGGA | GGGAAAGATAGTTGTGAATGAGC |
| PIK3CAEx12 | GGCAGTGTTTTAGATGGCTCA | CAAATCAGGGTCAGTTTCTGC |
| PIK3CAEx13 | GGTTCGAGGTTTTGCTGTTC | GGAAAACTCTTCCAGCCAAA |
| PIK3CAEx14 | CAGGAACTACCTGAAACTCATGG | CACAATGGCCTTATGAAGCA |
| PIK3CAEx15 | TCTGAGTGTTGCTGCTCTGTG | TTTGAGGGTAGGAGAATGAGAGA |
| PIK3CAEx16 | GGATTCCTAAATAAAAATTGAGGTGA | TGCATATTTCAAAGGTCAAGACA |
| PIK3CAEx17 | CATGTGATGGCGTGATCC | GGTGACACTCCAGAGGCAGTAG |
| PIK3CAEx18 | GGGAAAGGCAGTAAAGGTC | TCAAATATTTCAAAGGTTGAGCA |
| PIK3CAEx19 | TAAATGGAAACTTGCACCCTG | AAACAAATGGCACACGTTCTC |
| PIK3CAEx20 | TGGTGAAAGACGATGGACAAG | TGAGCTCAAGTGATCCTCCA |
| PIK3CAEx21 | GACATTTGAGCAAAGACCTGAAG | TGGATTGTGCAATTCCTATGC |
| FRAP1Ex44 | TGCTGTGCACTTCCTCTGAC | TGCTCTGTGACCTCCATCAG |
| FRAP1Ex45 | CGGGCTCCTGAGGAATATCT | TGCCTCCAGGGAAGAATTTA |
| FRAP1Ex46 | TCCTGGCAGGGTTAACTGTC | GGGAGAAGTGGGTGACAGAA |
| FRAP1Ex47 | TTGAATGCAGTGGTGCTCTC | TGCCCAGCCTTTTTCTTCTA |
| FRAP1Ex48 | TCTGCCTGTGTTCTGAGCTG | TCCCTAGGATGGTGAAAACAA |
| FRAP1Ex49 | CGTTTCTCCTTTTGCCATGT | TGTCTTGCTCACCCATTTCA |
| FRAP1Ex50 | AGCCAAGATAGCACCACTGC | TGGCATCACAATCAATAGGG |
| FRAP1Ex51 | CTCTGCTGTCGTAGCATGGA | AGGTGGTATGGAGGGTAGGG |
| FRAP1Ex52 | AATCAGTGCAGGTGATGCAG | AGAAGGAATCAGGGCAGGAC |
| FRAP1Ex53 | GTGAGTGGCTCTGTCCCATT | CAGCCAAGCAGAACTGTGAG |
| FRAP1Ex54 | CCCACCCACTTATTCCTGAG | TAACAAAGCCCATCCCATTC |
| FRAP1Ex55 | TAGGTAGGGCAGGCGTTAAA | GGTGCCCTGTTTTTCTCAAA |
| FRAP1Ex56 | CCTGAGAGGGGTCAACAAGA | GTGCCAAAGCTCGTCACTAA |
| FRAP1Ex57 | GGCCAAACTTTTCAAATCCA | ACAATGGGCACATGCAGTAA |
| AKT3Ex1 | TTGCTCTGTGAGTTGCAAGAA | CACGCTACACACAACATACCAG |
| AKT3Ex2 | ATTTTTGGAGGCCAGTGTTG | CGATAACCTCTATGCTAAGGGACT |
| AKT3Ex3 | GTTCGCACTGGGTTTTGTTT | TTCTAGCATGCCCAGAGGTT |
| AKT2Ex2 | GCCTTACCCTTTGCTGAGTG | GTCAGATCCCTGCTCTCCTG |
| AKT2Ex3 | TTGTGAGTCACCGTCACACTG | GCTCAATGACCAAGTCCCAC |
| AKT2Ex4 | GGCTTCTCTCCTTCCACACAC | CCAGAAGCGCAGATAGGAAAC |
| AKT2Ex5 | CCCTTCAGCTCTCTCTGGTTC | AGGGCAGCCTTGTCTCTCAG |
| AKT1Ex3 | GAGAGCTTAGAGGGATGGCAG | AGGCACTCACAGACCCTGG |
| AKT1Ex4 | CCTAAGAAACAGCTCCCGTACC | AGCCAGTGCTTGTTGCTTG |
| AKT1Ex5 | TTGGAGAGAGGAAGAGATGGG | AGTGAGGATGGCTACAGGCAG |
| AKT1Ex6 | GTGGAACCACGCTTGTGAG | GTGGAGTGCTGAGTGTCTCCT |
